# Models and Experiments in Bioengineering: Why Synergies Are Encouraged

**DOI:** 10.3389/fbioe.2015.00207

**Published:** 2016-01-11

**Authors:** Andrea Malandrino

**Affiliations:** ^1^Institute for Bioengineering of Catalonia, Barcelona, Spain; ^2^Department of Mechanical Engineering, Massachusetts Institute of Technology, Cambridge, MA, USA

**Keywords:** modeling and simulations, *in vitro* model, collaborative research, *in silico* modeling, synergies

Does the future of bioengineering lie in models or experiments? The long-standing debate on how and how much experimental and theoretical research in our field should cooperate can shape the future. This debate means often a choice, early in the bioengineer’s career. A bioengineer may prefer to address scientific questions based on experimental tests, or on computational and theoretical analyses. This fundamental choice can drive the future bioengineer’s mindset. Unsurprisingly, laboratories and researchers often assemble in separate, competitive teams. Indeed, the way bioengineers tackle a question is shaped by the characteristic techniques – experimental, theoretical, and computational – they are trained for. Here, without neglecting the obvious advantages of specializing exclusively in experimental, or theoretical and computational studies, I advocate a way of doing synergistic research that combine different approaches. Moreover, I believe that bioengineering desperately needs synergy and cooperation to pave the way for significant discoveries. We need efforts to train a new generation of biomedical engineers: individuals who take advantage of these synergies, who design projects accordingly, and who make these approaches work cooperatively.

## Do We Need all Modelers?

In biomedical engineering, research projects and careers are often shaped depending on the *modeling* approach used to investigate the human body: (i) models that use animals, called *in vivo* approaches; (ii) models in which tissues, cells, and molecules are tested outside their normal environment, called *in vitro* approaches; and (iii) theoretical models and computer simulations of biological processes, the so-called *in silico* approaches. Traditionally, experimentalists are those who chose the *in vivo* or *in vitro* modeling, whereas theoreticians or computational modelers are those who chose the *in silico* modeling. Yet, all the techniques in all three approaches are tied to the concept of the “modeling of the human body.” Clearly, the level of abstraction, the control of parameters, and the typical tools and backgrounds differ depending on a particular modeling approach. Also, additional modeling approaches to the simplified scheme proposed above, such as surrogate physical models (Roberts et al., 2012) have proved very effective in bioengineering research. It is now increasingly accepted that all of these models are equally needed, serving complementary purposes. There is also a need for multifaceted bioengineering modelers, who can pick the right approach at the right time for a given research question, and who can switch expertly among *in vivo*, *in vitro*, and *in silico* models to eventually merge different facets and find an answer. For instance, in systems biology, continuous feedback between extensive computing technologies and quantitative empirical data are paramount in the discovery of new drugs, disease genes, emergent behaviors, and relevant biological pathways in human development and disease (Chuang et al., 2010). Researchers with direct experience in merging and coordinating experimental, theoretical, and computational modeling can ultimately accelerate discoveries and advantages from this feedback.

## Do We Need all Experimentalists?

Advantages from feedback between experimentalists and theoreticians can be undoubtedly rich, and even richer if we relax the strict borders of each group. For instance, validation is an important prerogative for a theoretical model. Whenever possible, validation must be accomplished by accompanying and challenging the model with independent, well-designed experiments. Nevertheless, new hypotheses and refined experiments are often generated after the use of a theoretical model to explain empirical data. Fascinatingly, the history of science abounds of theoretical speculations anticipating phenomena that have been later verified by experiments. For instance, Turing patterns, hypothesized by the well-known mathematician Alan Turing in the context of morphogenesis (Turing, 1952), were reproduced experimentally decades later with a dedicated experimental device (Ouyang and Swinney, 1991). Examples of Turing patterns in morphogenesis are emerging and being accepted in biology [see, for instance, Raspopovic et al. (2014)].

Bioengineering and biophysical approaches to significant discoveries in human development and disease face interdisciplinary and complex scenarios and must seek synergic assistance. For instance, advanced computational procedures are hoped to promote what could be called “*in silico* experiments.” These computational simulations may serve multiple purposes, such as making an initial selection of parameters or conducting further research. Indeed, a first test with such an *in silico* experiment with a high control on a large number of parameters will make refinements of further *in vitro* and *in vivo* experiments more effective and targeted. Concomitantly, in the era of *big data*, bioengineers are increasingly designing physiologically relevant *in vitro* tests with a high-throughput and high level of control. Similarly to a computational model, these high-throughput devices allow analysis of a larger amount of data produced with tools that were traditionally tied to multi-parametric, simulation studies (de Boer and van Blitterswijk, 2013). Again, these collaborative research efforts and the consequent discoveries will be accelerated by researchers who have been trained in the importance of a continuous interplay among all methodologies for hypothesis testing.

## A Horizon of Interplays and Synergies

The interplays and synergies among modeling and experimental approaches are increasingly used by a new generation of bioengineers. This generation is capable of transferring methodologies, visions, and mindsets of experiments into models, and of models into experiments. This generation is capable of dissolving boundaries and merging facets of both traditions. Such researchers will test a computational model for its reliability by comparison with experimental models, and by coordinating and refining *in vivo* or *in vitro* tests based on *in silico* assessments. These researchers should operate in search of synergies, collaborating with different groups, and aiming at studies where the interplay between these laboratories and computational techniques is encouraged. This generation will ultimately translate synergic efforts into innovative research projects. The focus of the study might pass from choosing among *in vivo*, *in vitro*, or *in silico*, to “how to coordinate all of them.” This evolution is not straightforward, but researchers who understand and orchestrate approaches from all sides will produce results with wider spectra of interpretations, whether these results show emergent behaviors, new phenomena, or explanations for observations. Similarly, contingency plans after failure of a given project task can be more effective if comprehensive judgments are made in light of these interplays. Ultimately, I believe that new research avenues are most likely to arise when the synergies are employed.

Let me finish this opinion essay with a personal anecdote. When starting my Master’s thesis research, I was asked if I preferred to have a computationally or an experimentally based project. I replied: could I do something in between? The answer was: it might be ambitious, but of course we can think about it. I continue to promote this approach.

## Author Contributions

AM conceived the work and wrote the paper.

## Conflict of Interest Statement

The author declares that the research was conducted in the absence of any commercial or financial relationships that could be construed as a potential conflict of interest.

## References

[B1] Chuang H.-Y. Hofree M. Ideker T. (2010). A decade of systems biology. Annu. Rev. Cell Dev. Biol. 26, 721–744. 10.1146/annurev-cellbio-100109-104122 20604711 PMC3371392

[B2] de Boer J. van Blitterswijk C. A. (2013). Materiomics: High-Throughput Screening of Biomaterial Properties. Cambridge: Cambridge University Press.

[B3] Ouyang Q. Swinney H. L. (1991). Transition from a uniform state to hexagonal and striped Turing patterns. Nature 352, 610–612. 10.1038/352610a0

[B4] Raspopovic J. Marcon L. Russo L. Sharpe J. (2014). Modeling digits. Digit patterning is controlled by a Bmp-Sox9-Wnt Turing network modulated by morphogen gradients. Science 345, 566–570. 10.1126/science.1252960 25082703

[B5] Roberts J. C. Harrigan T. P. Ward E. E. Taylor T. M. Annett M. S. Merkle A. C. (2012). Human head-neck computational model for assessing blast injury. J. Biomech. 45, 2899–2906. 10.1016/j.jbiomech.2012.07.027 23010219

[B6] Turing A. M. (1952). The chemical basis of morphogenesis. Philos. Trans. R. Soc. Lond. B Biol. Sci. 237, 37–72. 10.1098/rstb.1952.0012 PMC4360114 25750229

